# *Leishmania (Leishmania) amazonenis* Infection, Suriname

**DOI:** 10.3201/eid1405.070433

**Published:** 2008-05

**Authors:** Wendy van der Meide, Henry de Vries, Francine Pratlong, Allard van der Wal, Leslie Sabajo

**Affiliations:** * Royal Tropical Institute, Amsterdam, the Netherlands; † Academic Medical Center, Amsterdam, the Netherlands; ‡ Health Service Amsterdam, Amsterdam, the Netherlands; § Centre National de Référence des *Leishmania* and Université Montpellier, Montpellier, France; ¶ Dermatology Service, Paramaribo, Suriname

**Keywords:** Leishmania L. amazonenis, Suriname, miltefosine, letter

**To the Editor:** A 17-year-old man was seen at the Dermatology Service in Paramaribo (Suriname) with a skin condition that he had had since he was 5 years of age. The condition consisted of multiple cutaneous ulcerations, nodules, and fibrotic plaques disseminated on his face, limbs, and trunk, and subcutaneous nodules on lymph-draining tracts on his hands, arms, and legs (Appendix Figure, panel** A**). He had lived his entire life in an inland village, located at Brokopondo Lake (central-eastern Suriname); he had never traveled outside the country. The diagnosis of cutaneous leishmaniasis (CL, a parasitic disease caused by the protozoa *Leishmania*) was presumed. The patient received pentamidine therapy in 1997, 1998, and 2005, but without sustained clinical effect. Rapid screening tests for HIV were negative (Determine [Abbott Laboratories, Tokyo, Japan] and Unigold [Trinity Biotech, Co. Wicklow, Ireland]). In 2006, the diagnosis of CL was confirmed with histopathology, culture, and PCR. The parasite was identified by a PCR restriction fragment length polymorphism method on the small subunit–internal transcribed spacer genes (*1*) and by multilocus enzyme electrophoresis at the National Reference Center of *Leishmania* (Montpellier, France).

After promising results were obtained with miltefosine in a patient with anergic diffuse cutaneous leishmaniasis (ADCL) in Venezuela (*2*), the patient received 150 mg/day oral miltefosine (Impavido, Zentaris, Germany) for 98 days and the lesional parasite load was quantified with quantitative nucleic acid sequence-based amplification (*3*). Skin biopsy specimens were collected from 1 target lesion before treatment; during treatment at day 14, day 28, day 42 (all in duplicate); and at day 70 (single biopsy).

The strain causing infection (MHOM/SR/2006/SP100) was identified as *Leishmania (Leishmania) amazonensis,* and the enzymatic profile was equal to *L. (L.) amazonensis* zymodeme MON-41. Histopathology showed large macrophages containing abundant *Leishmania* amastigotes and few lymphocytes and plasma cells without granuloma formation. A considerable clinical improvement was observed during the first 2 months of therapy. The lesions slowly decreased in size and duration. At day 70, all ulcerative lesions were re-epithelialized, without signs of infiltration or lymphangitis (Appendix Figure, panel** B**). At start of treatment, parasite counts of 360,000 and 310,000 parasites per biopsy were detected; these counts decreased to 0 parasites/biopsy at day 70. Histopathologic studies at day 70 showed no *Leishmania* bodies, a dense lymphocytic and plasma cellular infiltrate, and fibrosis. Apart from mild elevation of creatine and urea during treatment, no subjective or adverse side effects were reported.

*L. (L.) amazonensis* causes CL and 2 very serious manifestations of CL, disseminated cutaneous leishmaniasis (DCL) and ADCL (*4*). Both forms are histopathologically characterized by heavily parasitized macrophages and an absence of cell-mediated immune responses in therapy-naive patients (*4*). ADCL is resistant to any form of therapy, and cell-mediated immune responses never seem to occur. In contrast, the cell-mediated immune response in DCL can eventually arise upon therapy response, even in patients with previous therapy failures (*4*). The therapy response in DCL patients is histopathologically characterized by the appearance of a lymphocytic and plasma cellular infiltrate. The diagnosis of DCL is plausible in our patient based on the histopathologic findings before, during, and after therapy; the clinical picture (erythematous infiltrated plaques, lymphadenitis, and lymphangitis), and the favorable therapy response. He was last seen 7 months after end of therapy, at which time new lesions had not developed.

In general, *L. (L.) amazonensis* infection is rare in humans (*5*). In French Guiana, bordering the eastern side of Suriname, few patients (≈1.9%) are reported to be infected with this species (*5*). However, the sandfly vector of *L. (L.) amazonensis*, *Lutzomyia flaviscutellata,* was detected earlier in Suriname (*6*), which may indicate transmission of *L. (L.) amazonensis* infection to humans by means of the bite of this sandfly in Suriname. Our patient had no history of transfusion or intravenous drug use.

Many gold diggers from the northern part of Brazil work and travel in Suriname and are familiar with CL. In the Brazilian State Pará, a region bordering Suriname in the South, the infection rate with *L. (L.) amazonensis* is high (34.8%) (*7*). It is thus conceivable that infected gold diggers from that area have introduced *L. (L.) amazonensis* into Suriname. Our patient used to live in a village where many Brazilian gold diggers worked around the time that his skin lesions developed. Migration of laborers is associated with an increased risk for CL infection (*8*). The zymodeme MON-41 is widespread in Central America and the northern part of South America, and has been reported in Venezuela, Brazil, Panama, French Guiana, and Colombia (F. Pratlong and J.P. Dedet, Montpellier International Cryobank of *Leishmania*, pers. comm., 2007). Therefore, speculations on the exact origin of the infection need to be made cautiously.

## Supplementary Material

Appendix Figure Infiltrated lesions on the patient's hands A) before and B) 70 days after treatment began.
