# Supplementary figures and images for: Leishmania (Leishmania) amazonenis Infection, Suriname
Source: Emerg Infect Dis. 2008 May;14(5):857–9. doi: 10.3201/eid1405.070433 (PMC2600244; doi:10.3201/eid1405.070433)

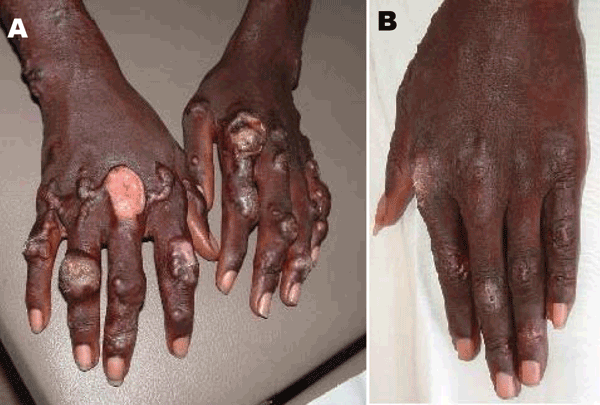

Supplement: Appendix Figure — Infiltrated lesions on the patient's hands A) before and B) 70 days after treatment began. [file 07-0433_app-s1.gif]
